# Help from the little guy: Intramural ischemic ventricular tachycardia ablation leveraging a 2F multipolar catheter in a coronary vein

**DOI:** 10.1016/j.hrcr.2024.05.021

**Published:** 2024-06-03

**Authors:** Jacob N. Blackwell, N. A. Mark Estes, Alaa A. Shalaby

**Affiliations:** Division of Cardiac Electrophysiology, Heart and Vascular Institute, University of Pittsburgh School of Medicine, Pittsburgh, Pennsylvania

**Keywords:** Ventricular tachycardia, Radiofrequency ablation, Multipolar ablation, Ventricular tachycardia mapping, Intramural arrhythmia substrate

**Key Teaching Points**

• We describe the use of a multielectrode mapping catheter placed in the anterior interventricular vein to localize and ablate an intramural, ischemic ventricular tachycardia (VT) circuit.
• The case demonstrates the novel use of multipolar ablation to create an extensive transmural lesion set in the treatment of ischemic, scar-mediated VT.
• Multipolar ablation of intramural ischemic VT via the coronary sinus system provides an opportunity to affect intramural VT isthmus sites and avoids the risks associated with pericardial access.

## Introduction

Radiofrequency catheter ablation has been established as an effective therapy for ventricular tachycardia (VT).^1^^,^^2^ Limitations of endocardial ablation approaches have been described, with intramural substrates representing a barrier to successful ablation.^3^^,^^4^ Bipolar ablation strategies have emerged as an option to achieve transmural lesions for intramural VT and premature ventricular complexes (PVCs) in certain anatomic regions.^1^^,^^5^, ^6^, ^7^, ^8^ Recent work has demonstrated the use of multipolar ablation via the coronary sinus system as a means of achieving transmural lesions for the treatment of PVCs.^9^

We describe the case of a patient with ischemic cardiomyopathy and recurrent, scar-mediated VT. The case demonstrates incorporation of a multipolar mapping catheter placed in the anterior interventricular vein for ablation of ischemic, scar-mediated VT.

## Case report

**Figure 1:**
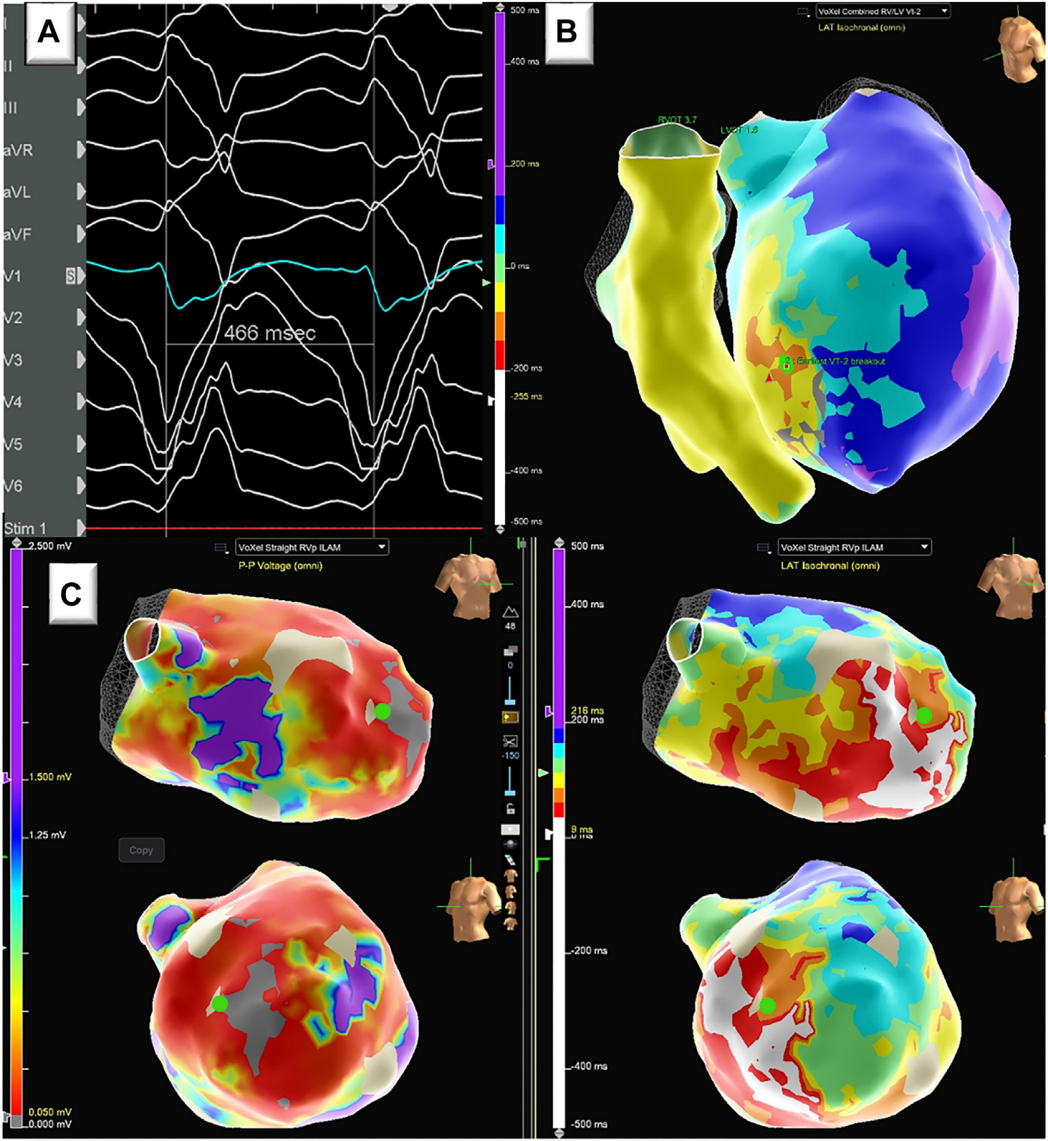
Clinical ventricular tachycardia (VT) and substrate. **A:** Twelve-lead electrocardiogram of apical anteroseptal VT. **B:** Combined left ventricular (LV) and right ventricular activation map encompassing 77% of the total cycle length, with earliest LV activation denoted by green dot. **C:** LV voltage map (1.5 mV–0.05 mV) and isochronal late activation map with earliest LV activation site denoted by green dot.

A 68-year-old male patient with ischemic cardiomyopathy, left ventricular ejection fraction 15%–20%, and recurrent monomorphic VT presented with progressive dyspnea and was found to be in sustained monomorphic VT and cardiogenic shock. The patient had previously undergone successful ablation of a left ventricular midseptal VT with a cycle length of 430 ms 3 months prior. Left ventricle (LV) endocardial as well as LV to right ventricle (RV) bipolar ablation were used. The presenting VT had a different morphology with a cycle length of approximately 466 ms (Figure 1A). His presentation was complicated by amiodarone-induced acute interstitial pneumonitis, which resolved with steroids. Following inotrope-supported diuresis, the patient was brought to the electrophysiology lab for VT ablation. Substrate mapping of the ventricles during RV pacing was performed using the EnSite mapping system and Advisor HD Grid mapping catheter (Abbott Laboratories, Chicago, IL) (Figure 1C). VT was readily induced with double extrastimuli programmed pacing. Seventy-seven percent of the cycle length was obtained with mapping both ventricles. Activation suggested VT origin was close to the site of previous septal ablation but more anterior apical (Figure 1B). Entrainment from the endocardial earliest site of activation displayed manifest fusion.

**Figure 2:**
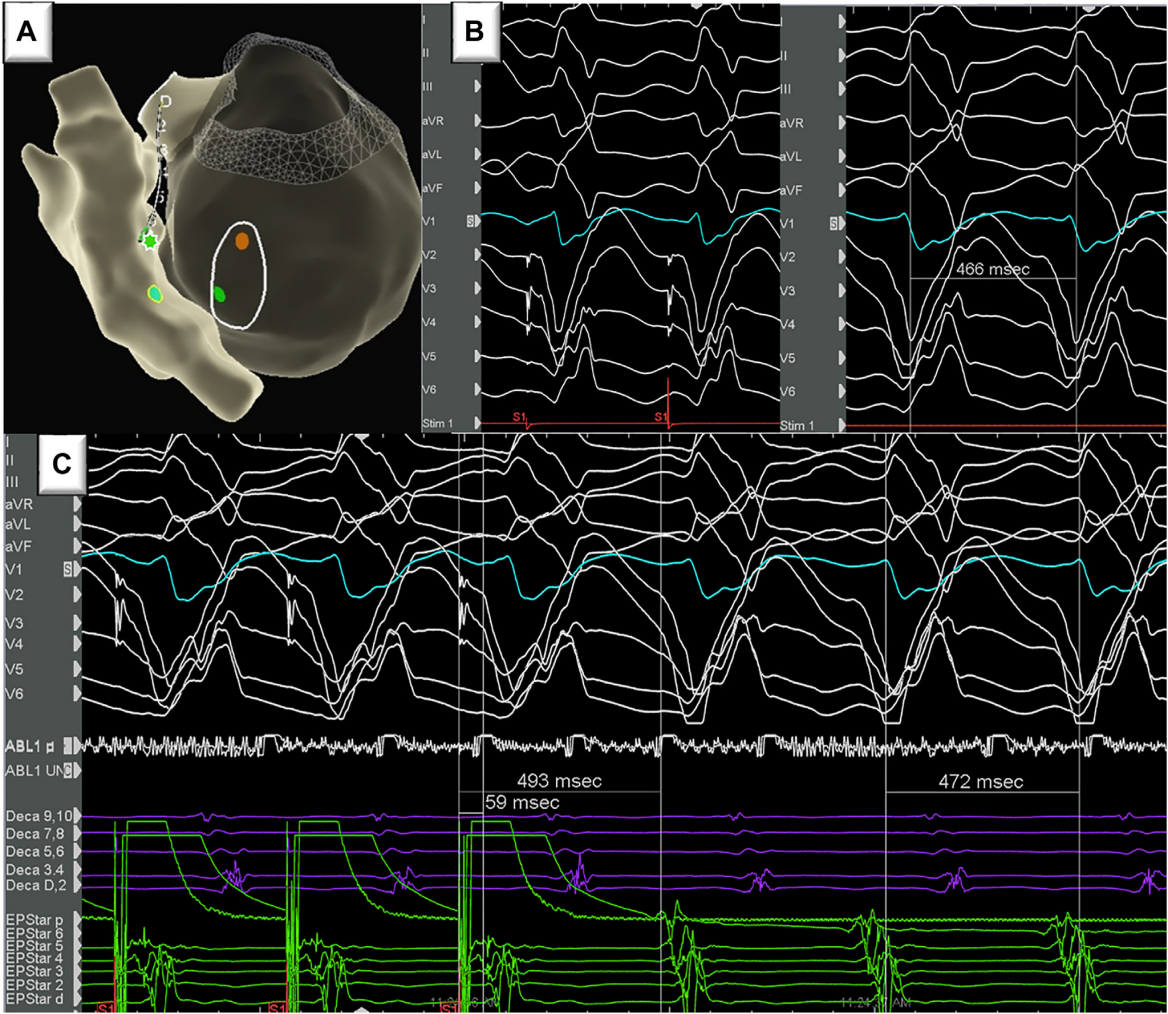
**A:** Anatomic shell depicting the areas of earliest activation for left ventricle and right ventricle (*green dot*) and anterior interventricular vein (AIV) (*green asterisk*). **B:** Pacing from earliest AIV activation site (*green asterisk*) via the distal multipolar catheter resulted in 98% morphologic match to clinical ventricular tachycardia (VT). **C:** Entrainment from AIV earliest activation site resulted in concealed fusion and was suggestive of VT exit site.

**Figure 3:**
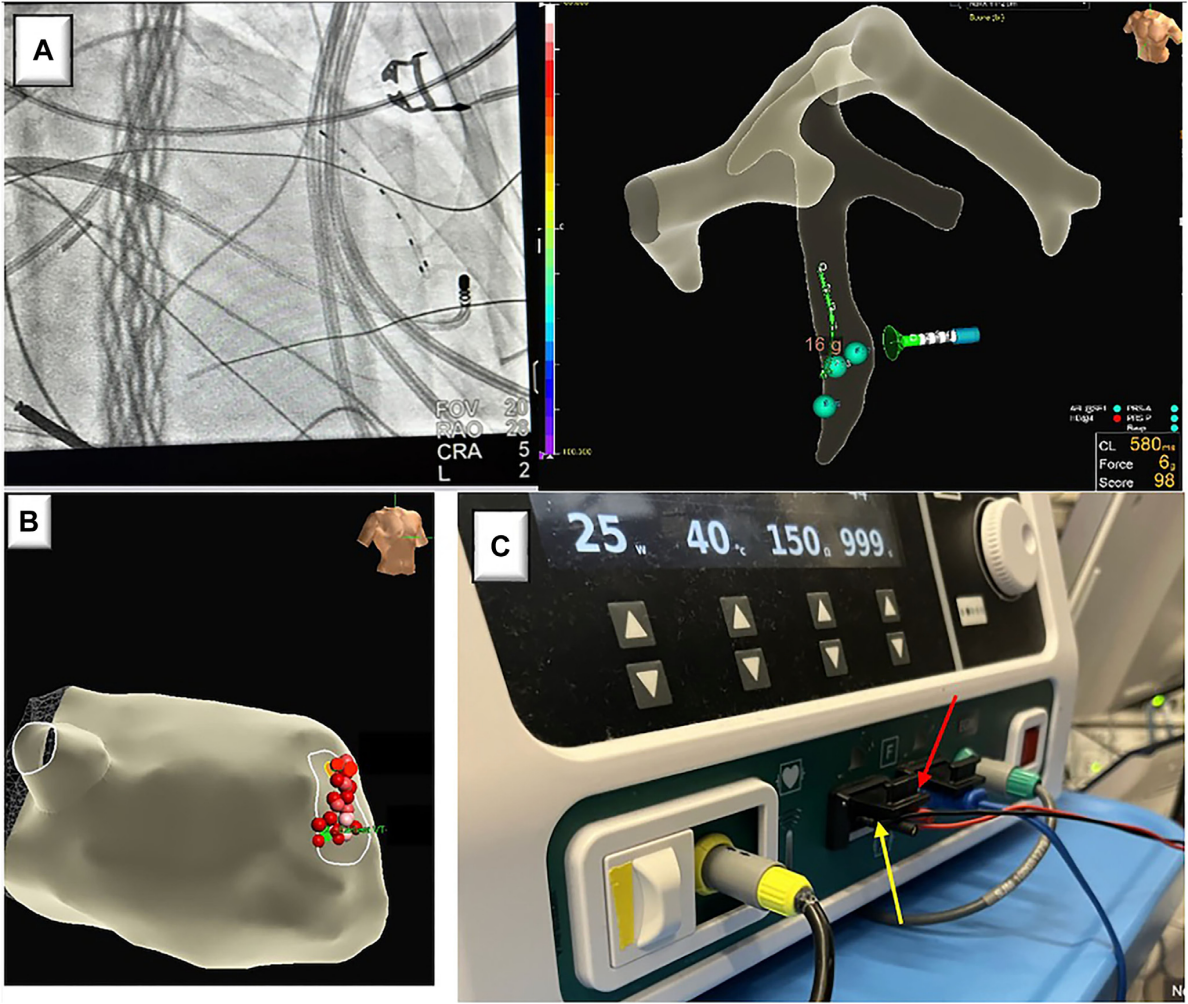
**A:** Radiographic and mapping system depiction of ablation vector between left ventricle (LV) endocardial ablation catheter and anterior interventricular vein–positioned multipolar catheter. **B:** Final lesion set created by adjusting the LV endocardial ablation catheter position in relation to the multipolar catheter. **C:** Generator setup for multipolar ablation with EPstar 6 (Baylis Medical) connected to the ampere return via the red jumper cable (*red arrow*) and EPstar 8 (Baylis Medical) connected to the ampere return via the black jumper cable (*yellow arrow*).

The coronary sinus was cannulated next with an Agilis short curve (Abbott Laboratories, Chicago, IL), and a 2F EPstar Fixed Electrophysiology Catheter (Baylis Medical) with 5 mm interpolar spacing was advanced into the distal anterior interventricular vein (AIV, Montreal, QC, Canada). Pace map at the site of earliest activation in the AIV was 98% congruent with the clinical VT (Figure 2B). Entrainment displayed concealed fusion from the distal AIV consistent with an exit site stimulus to QRS onset at 13% of cycle length (Figure 2C). Earliest activation from the RV, LV, and AIV were 32 ms, 40 ms, and 32 ms pre-QRS, respectively (Figure 2A). Multipolar ablation between the TactiFlex (Abbott Laboratories, Chicago, IL) at the earliest site on the LV endocardium and sequentially to the most distal poles (6-8) of the EPstar catheter was performed. Average starting impedance was 93 ohms. Lesions were delivered at 15–25 W (median of 20 W) for an average time of 51 seconds (20–60 seconds) (Figure 3) with an endpoint of endocardial electrogram abolition and lack of capture. Average contact force on the LV endocardium was 12*g* with an average impedance drop of 11% for the multipolar lesion set. Minor adjustments in ablation catheter position were made on the LV endocardium in the area of interest to modify the vector of ablation (Figure 3B). In view of known prior LAD occlusion and scar, we did not perform a coronary catheterization prior to delivering ablation lesions. After these ablation lesions were delivered, VT was no longer inducible, and high-output pacing at the site of ablation in the LV endocardium did not capture myocardium. At 3 months follow-up, he remains without recurrence of VT.

## Discussion

We report a case of a successful multipolar ablation of a scar-mediated, intramural monomorphic VT. This case demonstrates the use of a small-caliber, multipolar mapping catheter as the return in a radiofrequency ablation circuit for effectively eliminating an intramural ischemic VT.

Endocardial ablation techniques are limited by effective lesion depth. Epicardial access for ablation of intramural and epicardial portions of the critical VT isthmus can be effective in some cases, but is not possible in presence of prior coronary artery bypass graft and is limited by increased procedure times and risk of procedural complications.^2^^,^^10^^,^^11^ Utilization of the coronary sinus system and bipolar ablation offer a strategy for obtaining transmural lesions in the treatment of VT if the critical isthmus is close to venous structures.^5^, ^6^, ^7^ However, ablation catheter diameter and vein caliber can highly limit the ability to safely navigate and perform bipolar ablation in a site of interest. The small caliber of the multielectrode mapping catheter used in this case allows for access to distal branches of the coronary sinus system, thus increasing the area of possible ablation vectors. A force sensing ablation catheter in contact with the endocardium is the active arm of the ablation circuit with the small-diameter multielectrode mapping catheter functioning as the return. The small surface area of the 2 return electrodes on the mapping catheter and the proximity to the ablation catheter result in a high current density and transmural ablation. The biophysics of multipolar ablation lesions have been previously described in ex vivo and in vivo animal model studies.^9^

Multipolar ablation using the coronary sinus system has been described in the ablation of focal PVCs.^9^ In the presently described case, transmural ablation of a scar-mediated monomorphic VT was achieved in a distal septal location where a regular ablation catheter could not be advanced. By adjusting the position of the ablating catheter on the LV endocardium, we were able to modify the ablation vector, creating a region of transmural tissue ablation that encompassed the VT isthmus.

We believe our case highlights the utility of a novel approach to tackle VT circuits in close proximity to the coronary venous system. We recognize that bipolar ablation with a return ablation catheter placed in the pericardial space could have offered the same outcome. Pericardial access, however, has its own risks and limitations. Similarly, at this time we cannot report the extent of the transmural lesion delivered.

## Conclusion

Ischemic, scar-mediated VT not responsive to conventional endocardial ablation strategies can be successfully ablated using multielectrode mapping catheters placed in the coronary sinus system as a multipolar ablation circuit return. This approach allows for broader access to distal portions of the coronary venous system and adjacent myocardium compared to previously described bipolar ablation approaches. Large regions of transmural lesions can be created with this approach for ischemic VT ablation. Further clinical studies that incorporate cardiac imaging should be done to better elucidate the safety and efficacy of this novel ablation strategy.

## Disclosures

The authors of this manuscript have no conflicts of interest to disclose.

## References

[1] Zeppenfeld K., Tfelt-Hansen J., De Riva M. (2022). 2022 ESC Guidelines for the management of patients with ventricular arrhythmias and the prevention of sudden cardiac death. Eur Heart J.

[2] Cronin E.M., Bogun F.M., Maury P. (2020). 2019 HRS/EHRA/APHRS/LAHRS expert consensus statement on catheter ablation of ventricular arrhythmias. Heart Rhythm.

[3] Stevenson W.G., Wilber D.J., Natale A. (2008). Irrigated radiofrequency catheter ablation guided by electroanatomic mapping for recurrent ventricular tachycardia after myocardial infarction: the multicenter thermocool ventricular tachycardia ablation trial. Circulation.

[4] Ghannam M., Liang J., Sharaf-Dabbagh G. (2020). Mapping and ablation of intramural ventricular arrhythmias: a stepwise approach focused on the site of origin. JACC Clin Electrophysiol.

[5] Koruth J.S., Dukkipati S., Miller M.A., Neuzil P., d’Avila A., Reddy V.Y. (2012). Bipolar irrigated radiofrequency ablation: a therapeutic option for refractory intramural atrial and ventricular tachycardia circuits. Heart Rhythm.

[6] Della Bella P., Peretto G., Paglino G. (2020). Bipolar radiofrequency ablation for ventricular tachycardias originating from the interventricular septum: safety and efficacy in a pilot cohort study. Heart Rhythm.

[7] Enriquez A., Hanson M., Nazer B. (2023). Bipolar ablation involving coronary venous system for refractory left ventricular summit arrhythmias. Heart Rhythm O2.

[8] Baszko A., Telec W., Kałmucki P. (2016). Bipolar irrigated radiofrequency ablation of resistant ventricular tachycardia with a septal intramural origin: the initial experience and a description of the method. Clin Case Rep.

[9] Fernandes G.C., Nguyen T., Creed E. (2023). Multipolar Ablation Using Mapping Electrodes. JACC Clin Electrophysiol.

[10] Sosa E., Scanavacca M., d’Avila A., Oliveira F., Ramires J.A.F. (2000). Nonsurgical transthoracic epicardial catheter ablation to treat recurrent ventricular tachycardia occurring late after myocardial infarction. J Am Coll Cardiol.

[11] Della Bella P., Brugada J., Zeppenfeld K. (2011). Epicardial ablation for ventricular tachycardia. Circ Arrhythm Electrophysiol.

